# Liver Stiffness Hinders Normalization of Systemic Inflammation and Endothelial Activation after Hepatitis C Virus (HCV) Eradication in HIV/HCV Coinfected Patients

**DOI:** 10.3390/vaccines8020323

**Published:** 2020-06-19

**Authors:** Beatriz Álvarez, Clara Restrepo, Marcial García, María A. Navarrete-Muñoz, María A. Jiménez-Sousa, Laura Prieto, Alfonso Cabello, Sara Nistal, Salvador Resino, Miguel Górgolas, Norma Rallón, José M. Benito

**Affiliations:** 1 Hospital Universitario Fundación Jiménez Díaz, 28040 Madrid, Spain; BAlvarez@quironsalud.es (B.Á.); LPrietoPe@fjd.es (L.P.); ACabello@fjd.es (A.C.); MGorgolas@fjd.es (M.G.); 2 HIV and Viral Hepatitis Research Laboratory, Instituto de Investigación Sanitaria Fundación Jiménez Díaz, Universidad Autónoma de Madrid (IIS-FJD, UAM), 28040 Madrid, Spain; clara.restrepo@hospitalreyjuancarlos.es (C.R.); marcial.garcia@hospitalreyjuancarlos.es (M.G.); maria.navarretemu@hospitalreyjuancarlos.es (M.A.N.-M.); 3 Hospital Universitario Rey Juan Carlos, 28933 Móstoles, Spain; sara.nistal@hospitalreyjuancarlos.es; 4 Unidad de Infección Viral e Inmunidad, Centro Nacional de Microbiología, Instituto de Salud Carlos III, Majadahonda, 28040 Madrid, Spain; jimenezsousa@isciii.es (M.A.J.-S.); sresino@isciii.es (S.R.)

**Keywords:** systemic inflammation, endothelial activation, HIV/HCV coinfection, liver stiffness, HCV eradication, DAAs

## Abstract

Systemic inflammation, endothelial dysfunction and coagulopathy are of high clinical relevance in the management of people living with HIV (PLWH), and even more in patients coinfected with hepatitis C virus (HCV). It has been suggested a significant impact of HCV coinfection on these conditions. However, HCV can be eradicated in most patients with the new direct-acting antivirals (DAAs) therapy. We have analyzed the effect of HCV on systemic inflammation, endothelial activation and coagulopathy in PLWH and its evolution after HCV eradication with DAAs. Twenty-five HIV/HCV coinfected (HIV/HCV group), 25 HIV monoinfected (HIV group) and 20 healthy controls (HC) were included in the study. All patients were on ART and HIV suppressed. Levels of fourteen markers of systemic inflammation, endothelial activation and coagulopathy (IL-1ß, IL-6, IL-12p70, IL-8, TNFα, D-dimer, Eotaxin, IL-18, IP-10, monocyte chemotactic protein-1 (MCP-1), plasminogen activator inhibitor-1 (PAI-1), TNFα receptor 1 (TNFR1), vascular cell adhesion molecule 1 (VCAM-1) and intercellular adhesion molecule 1 (ICAM-1)) were measured on plasma at baseline and after DAAs-mediated HCV eradication. Non-parametric tests were used to establish inter/intra-group differences. At baseline, the HIV/HCV group showed increased levels of IL-18 (*p* = 0.028), IP-10 (*p* < 0.0001), VCAM-1 (*p* < 0.0001) and ICAM-1 (*p* = 0.045), compared to the HC and HIV groups, with the highest levels for IL18 and IP10 observed in HIV/HCV patients with increased liver stiffness (≥7.1 KPa). Eradication of HCV with DAAs-based therapy restored some but not all the evaluated parameters. VCAM-1 remained significantly increased compared to HC (*p* = 0.001), regardless of the level of basal liver stiffness in the HIV/HCV group, and IP-10 remained significantly increased only in the HIV/HCV group, with increased level of basal liver stiffness compared to the HC and to the HIV groups (*p* = 0.006 and *p* = 0.049, respectively). These data indicate that DAAs therapy in HIV/HCV co-infected patients and HCV eradication does not always lead to the normalization of systemic inflammation and endothelial dysfunction conditions, especially in cases with increased liver stiffness.

## 1. Introduction

Among people living with HIV (PLWH), the advent of combination antiretroviral therapy (cART) has reduced the incidence of morbidity and mortality, improving life expectancy [1]. Nevertheless, despite successful cART, there is evidence of the existence of a state of persistent systemic inflammation [2,3], and a heightened incidence of several comorbidities, including cardiovascular events, diabetes mellitus, kidney and liver diseases and cancer [3,4], which has been associated with that state of heightened inflammation [5]. On the other hand, in PLWH the existence of coinfection with hepatitis C virus (HCV) is relatively common, especially in some epidemiological groups mainly injected drug users, men who have sex with men and sex workers [6]. The existence of coinfection poses an additional stress on host immune system and, as a consequence, each virus can aggravate the normal clinical course of the concomitant infection [7,8].

The effect of HCV on different aspects of HIV disease pathogenesis has been explored, and previous studies have shown an increase in T-cells homeostasis disturbances in PLWH coinfected with HCV [9,10]. Furthermore, since HCV infection is also associated with a state of chronic inflammation [11], an additive effect of HCV co-infection in PLWH might be expected. In fact, previous studies have shown that patients coinfected with HIV and HCV present higher levels of soluble markers of systemic inflammation [12,13,14,15,16], monocyte activation [13,15,16] and endothelial dysfunction [12,16] when compared to HIV monoinfected patients. However, the relationship between inflammation, HCV and liver damage is not fully understood, although some studies suggest that the extent of liver damage (the degree of liver fibrosis and/or necroinflammatory activity) is associated with the level of systemic inflammation in HIV/HCV coinfected patients [16].

The effect of HCV coinfection on markers of systemic inflammation in PLWH has also been explored in cohorts of patients after HCV eradication with anti-HCV therapy. A few previous studies have addressed this, although only a few soluble markers were measured [17,18] or the studies were performed in patients treated with IFNα plus ribavirin [12], and thus, the results may be biased, due to the immunomodulatory effect of IFNα [19]. The current scenario of anti-HCV treatment with direct-acting antivirals (DAAs), showing rates of HCV eradication higher than 90% and devoid of immunomodulatory action [20], gives us the opportunity to evaluate the effect of HCV eradication on inflammation markers. In the present study, we have evaluated the effect of HCV on several soluble markers of systemic inflammation, coagulation and endothelial activation in PLWH coinfected with HCV, as well as their evolution after HCV eradication with DAAs therapy.

## 2. Materials and Methods

### 2.1. Study Participants and Sample Collection

This is a study with an observational, retrospective and longitudinal design. The study included a total of 50 patients with chronic HIV infection, 25 of them coinfected with HIV and HCV (HIV/HCV group) and 25 monoinfected with HIV (HIV group). All patients were recruited at the outpatient clinic of Hospital Universitario Fundación Jiménez Díaz in Madrid, Spain. At inclusion, all patients were on cART and undetectable plasma HIV-RNA load (<50 copies/mL), and had CD4 T-cell counts of at least 350 cells/µL. All HIV/HCV patients were naive for anti-HCV therapy at enrolment, and were candidates to be treated with DAAs, according to current national guidelines. Twenty age and sex-matched HIV and HCV seronegative healthy controls (HC group) from blood banking were also included, as a reference group for the inflammation markers evaluated. Blood samples were collected from all subjects included in the study. For HIV/HCV patients, one additional sample was collected 12 weeks after the end of treatment with DAAs when sustained virological response (SVR) is evaluated (SVR was defined as HCV RNA <15 IU/mL). Blood samples were collected in EDTA tubes, and cell-free plasma was aliquoted and stored at -20ºC until analysis. To participate in the study, written informed consent was obtained from all subjects and the study protocol was evaluated and approved by the Ethical Committee of Hospital Universitario Fundación Jiménez Díaz, Madrid, Spain (PIC 46/2015, record number 07/15 dated on April 14th 2015).

### 2.2. Liver Stiffness Assessment

Baseline (pre-DAAs treatment) and post-DAAs treatment liver stiffness (LS) was assessed in all HIV/HCV patients by transient elastography (TE) using a FibroScan^®^ 530, and the results were expressed in Kilopascals (KPa). The same trained operator performed all LS measurements. For each measurement 20 acquisitions were performed. Validation criteria for measurements were a success rate of at least 70% of acquisitions, and an interquartile range of less than 30% of the median value of all acquisitions. Using LS values, the next cut-off values were used as predictors of the Metavir scale of fibrosis: LS < 7.1 KPa for F0-F1 stages, LS between 7.1–9.4 KPa for F2 stage, LS between 9.5–12.4 KPa for F3 stage and LS ≥ 12.5 KPa for F4 stage of fibrosis. Patients were stratified into two groups using the cut-off value of 7.1 KPa: patients with LS < 7.1 KPa (F0-F1) and patients with LS ≥ 7.1 KPa (F2-F4), according to a clinically relevant cut-off previously proposed [21].

As additional surrogate markers of liver disease, the aspartate aminotransferase to platelet ratio index (APRI) and Fibrosis-4 (FIB-4) index were calculated using the following formulae:

APRI: [AST (IU/L)/AST upper limit of normal (IU/L)] × 100/platelets (10^9^/L)

FIB-4: [age (years) × AST (IU/L)]/[platelet count (10^9^/L) × square root of ALT (IU/L)]

### 2.3. Measurement of Plasma Markers of Inflammation, Pro-Coagulation and Endothelial Activation

Levels of fourteen different plasma markers were measured using Procarta Plex multiplex immunoassay (Invitrogen, Thermo Fisher Scientific) following the manufacturer’s specifications, using a Luminex 200 analyzer (Luminex Corporation, Texas, USA) and Bio-Plex manager software (Bio-Rad, California, USA). The following markers were assessed: (a) pro-inflammatory markers: interleukins (IL)-1ß, IL-6, IL-12p70, IL-8, IL-18, tumor necrosis factor alpha (TNFα), TNFα receptor 1 (TNFR1), monocyte chemotactic protein-1 (MCP-1), interferon gamma-induced protein 10 (IP-10) and Eotaxin; (b) endothelial activation markers: intercellular adhesion molecule 1 (ICAM-1) and vascular cell adhesion molecule 1 (VCAM-1); (c) coagulopathy markers: D-dimer, and plasminogen activator inhibitor-1 (PAI-1).

Three separate multiplex panels were used to measure the fourteen different plasma markers: a 5-plex assay for IL-1ß, IL-6, IL-12p70, IL-8 and TNFα; a 7-plex high-sensitive assay for D-dimer, Eotaxin, IL-18, IP-10, MCP-1, PAI-1 and TNFR1; and a 2-plex assay for VCAM-1 and ICAM-1. All plasma samples were tested in duplicate.

### 2.4. Statistical Analysis

Different characteristics of the study groups are expressed as median [interquartile range, IQR] for continuous variables, and as percentage for categorical variables. Differences between the groups were tested using the nonparametric Kruskall-Wallis test and the Mann-Whitney U Test for continuous variables; and the Chi-squared test or Fisher‘s exact test for categorical variables.

Levels of plasma markers are reported as median [IQR]. Inter-groups comparisons were performed using the Kruskall-Wallis test (for multiple comparisons) and the Mann-Whitney U test (for pair-wise comparisons) tests. Intra-group comparisons (at baseline and after DAAs treatment in the HIV/HCV group) were performed using the Wilcoxon matched-pair rank test. Bivariate correlations were assessed using the Spearman’s rank correlation coefficient. A linear regression analysis using a stepwise selection method was performed to ascertain the factors associated with the levels of plasma markers. Statistical analyses were performed using SPSS software version 15 (SPSS Inc., Chicago, IL, USA). All *p*-values were considered significant when <0.05.

## 3. Results

### 3.1. Characteristics of Patients Included in the Study

The baseline clinical characteristics of HIV and the HIV/HCV groups of patients are summarized in Table 1. At enrolment, all patients were virologically suppressed with cART. There were no significant differences between patient’s groups on age, gender, time since HIV diagnosis, time on cART, CD4 T-cell counts, CD4/CD8 ratio, body mass index (BMI) and HIV transmission route. The group of healthy controls (HC) was matched with the groups of patients for age (46 (43–51) years, *p* = 0.175 for the global comparison between the three study groups) and for gender (100% of HC donors were male, *p* = 0.24 for the comparison between the HC and HIV groups). Seventy two percent of HIV/HCV patients carried HCV genotype 1 and 28% HCV genotype 4. Length of HCV infection was 2 (1.5–5.0) years and the HCV-RNA load was 6.1 (5.8–6.4) log copies/mL. Liver stiffness (LS) was <7.1 KPa in 60% (15 out of 25) and ≥7.1 KPa in 40% (10 out of 25) of HIV/HCV patients. Among the 10 patients with LS ≥ 7.1 KPa, all except two had LS values in the range of 7.1–9.4 KPa (F2 of the Metavir scale) (Table S1).

As expected, the HIV/HCV patients showed higher levels (IU/L) of ALT and AST (74 (49–162) and 58 (37–122), respectively) than the HIV patients (32 (24–37) and 27 (22–31), respectively; *p* < 0.0001 for both comparisons). In concordance with this, the APRI score was significantly higher in HIV/HCV patients compared to HIV patients. In contrast, the levels of total cholesterol, LDL and triglycerides were lower in the HIV/HCV patients (157 (125–177), 85 (71–104) and 95 (70–140) mg/dL, respectively) compared with the HIV patients (194 (162–227), 119 (93–146) and 138 (88–208) mg/dL, respectively, *p* < 0.0001, *p* = 0.001 and *p* = 0.050, respectively) (Table 1).

### 3.2. HCV Coinfection and Liver Stiffness Significantly Impacts on Markers of Inflammation and Endothelial Activation

To test the impact of HCV coinfection on the levels of plasma markers, we first compared the levels of these markers between the different groups of study (HC donors, HIV group and HIV/HCV group at baseline (before initiation of anti-HCV treatment, pre-DAAs HIV/HCV group).

**Figure 1:**
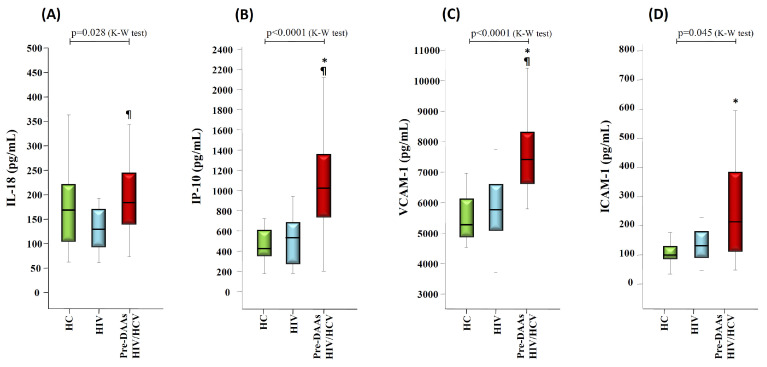
Box-plots graphs showing the levels of IL-18 (**A**), IP-10 (**B**), vascular cell adhesion molecule 1 (VCAM-1) (**C**) and intercellular adhesion molecule 1 (ICAM-1) (**D**) in healthy controls (HC) donors, HIV monoinfected (HIV), and in HIV/ hepatitis C virus (HCV) coinfected patients before direct-acting antivirals (DAAs) treatment (pre-DAAs HIV/HCV). *p*-values for global comparison between the groups (Kruskal-Wallis test, K-W) are shown. (*): *p* < 0.05 with respect to HC donors (Mann-Whitney U test). (**¶**): *p* < 0.05 with respect to the HIV group (Mann-Whitney U test).

First, the Kruskall-Wallis test showed significant differences between the three study groups for IL-18 (*p* = 0.028), IP-10 (*p* < 0.0001), VCAM-1 (*p* < 0.0001) and ICAM-1 (*p* = 0.045) with the highest levels observed in the pre-DAAs HIV/HCV group (Figure 1). Moreover, the pair-wise comparison (Mann-Whitney U test) showed significantly higher levels in pre-DAAs HIV/HCV compared to the HIV group for IL-18 (*p* = 0.008), IP-10 (*p* < 0.0001) and VCAM-1 (*p* < 0.0001) with a clear trend for ICAM-1 (*p* = 0.067). Similar results were observed when comparing the pre-DAAs HIV/HCV group of patients with HC donors, except for IL-18 (Figure 1).

**Figure 2:**
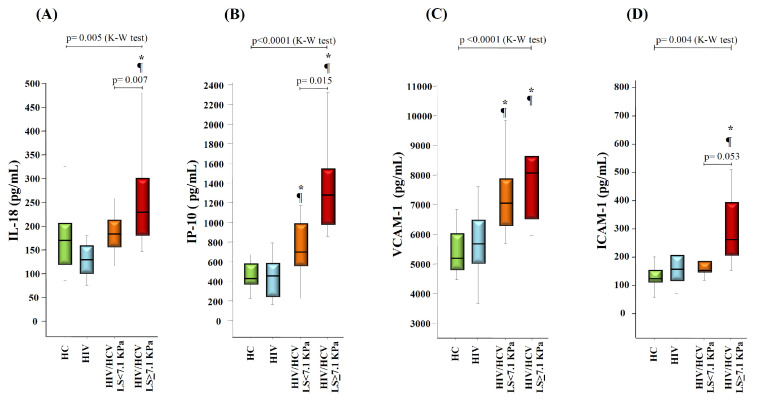
Box-plots graphs showing the levels of IL-18 (**A**), IP-10 (**B**), VCAM-1 (**C**) and ICAM-1 (**D**) in HC donors, HIV monoinfected, and HIV/HCV coinfected patients at baseline (before DAAs treatment) stratified according to level of liver stiffness (LS). *p*-values for global comparison between the groups (Kruskal-Wallis test, K-W) and for the comparison between the two groups of HIV/HCV patients (Mann-Whitney U test) are shown. (*): *p* < 0.05 with respect to HC donors (Mann-Whitney U test). (**¶**): *p* < 0.05 with respect to the HIV group (Mann-Whitney U test).

Next, to evaluate the impact of liver stiffness (LS) on these markers, we analyzed the levels of baseline plasma makers stratifying the HIV/HCV group, according to the level of LS at baseline (pre-DAAs therapy). Since most patients had LS values either below 7.1 KPa or in the range of 7–1-9.4 KPa, we stratified the patients in only two groups: patients with LS < 7.1 KPa (*n* = 15) and patients with LS ≥ 7.1 KPa (*n* = 10). The Kruskall-Wallis test showed significant differences between the four groups (HC donors, HIV group, and HIV/HCV groups according to level of LS) for IL-18 (*p* = 0.005), IP-10 (*p* < 0.0001), VCAM-1 (*p* < 0.0001) and ICAM-1 (*p* = 0.004), with the highest levels observed in HIV/HCV patients with LS ≥ 7.1 KPa (Figure 2). HIV/HCV patients with LS ≥ 7.1 KPa displayed higher levels of IL-18, IP-10, VCAM-1 and ICAM-1 when compared with the HIV group (*p* = 0.001, *p* < 0.0001, *p* < 0.0001 and *p* = 0.002, respectively) and HC donors (*p* = 0.014, *p* < 0.0001, *p* < 0.0001, and *p* = 0.001, respectively). In contrast, HIV/HCV patients with LS < 7.1 KPa showed significant increases only for IP-10 (*p* = 0.002 and *p* = 0.009 compared to HC donors and the HIV group, respectively) and for VCAM-1 (*p* < 0.0001 and *p* = 0.001, respectively). Moreover, higher levels of IL-18 (*p* = 0.007), IP-10 (*p* = 0.015) and ICAM-1 (*p* = 0.053) were observed in HIV/HCV patients with LS ≥ 7.1 KPa compared to those with LS < 7.1 KPa (Figure 2). In agreement with these findings, we also found a positive correlation of LS values with baseline plasma levels of IL-18 (rho = 0.463, *p* = 0.02), IP-10 (rho = 0.576, *p* = 0.003) and ICAM-1 (rho = 0.541, *p* = 0.046) (Figure S1).

### 3.3. Liver Stiffness Correlates with Markers of Liver Disease Severity

The potential correlation of LS values with different biochemical and immune-virological parameters at baseline was assessed. The parameters included in the correlation analysis were: liver enzymes, APRI score, FIB-4 index, lipid profile (triglycerides, total cholesterol, HDL and LDL), CD4 counts, CD4/CD8 ratio and HCV viral load. LS values were significantly correlated with levels of ALT, AST and GGT liver enzymes (*p* = 0.006, *p* = 0.001 and *p* = 0.021, respectively) (Figure S2). LS values also were significantly correlated with both APRI score (*p* = 0.001) and FIB-4 index (*p* = 0.001) (Figure S2). In contrast, there was no significant correlation with the rest of parameters tested.

### 3.4. Correlations between Baseline Levels of IL-18, IP-10, VCAM-1 and ICAM-1 with Markers of Liver Damage and with Virological Parameters

We also explored the potential correlations between plasma levels of IL-18, IP-10, VCAM-1 and ICAM-1, with parameters of liver damage, including the ALT, AST, GGT, APRI score and the FIB-4 index, and with levels of HCV replication. In Spearmans’s correlation analyses, we observed a significant positive correlation of IL-18 with ALT (rho =0.539, *p* = 0.005), with AST (rho = 0.461, *p* = 0.02), and with the APRI score (rho = 0.565, *p* = 0.003). A similar correlation profile was observed for VCAM-1 (ALT: rho = 0.641, *p* = 0.001; AST: rho = 0.562, *p* = 0.003; APRI score: rho = 0.582, *p* = 0.002), and for IP-10 levels (ALT: rho = 0.575, *p* = 0.003); AST: rho = 0.516, *p* = 0.008; APRI score: rho = 0.444, *p* = 0.026) that were also correlated with, GGT (rho = 0.465, *p* = 0.019). ICAM-1 was correlated only with APRI score (rho = 0.556, *p* = 0.039) and with the FIB-4 index (rho = 0.622, *p* = 0.018). Regarding the correlations with HCV replication level, only IL-18 was correlated with the HCV-RNA load (rho = 0.542, *p* = 0.005) (Figure S3).

Next, a linear regression analysis was performed to ascertain which factors were significantly and independently associated with the baseline plasma levels of IL-18, IP-10, VCAM-1 and ICAM-1. For each dependent variable (IL-18, IP-10, VCAM-1, ICAM-1), predictive variables included in the linear regression model were those with a significant association in the bivariate correlations: levels of liver enzymes, APRI score, FIB-4 index, HCV-RNA load and LS as a dichotomic variable (<7.1 or ≥7.1 KPa). The results of the linear regression models showed that: (a) the levels of IL-18 were associated with AST levels (β= 0.71 ± 0.14, *p* < 0.0001) and with HCV-RNA levels (β= 47.9 ± 18.3, *p* = 0.016); (b) the levels of IP-10 were associated with GGT levels (β= 1.60 ± 0.45, *p* = 0.002) and with LS ≥ 7.1 KPa (β= 509 ± 179, *p* = 0.01); (c) the levels of VCAM-1 were associated with AST levels (β= 5.78 ± 2.42, *p* = 0.025) (Table 2).

### 3.5. Changes in Levels of Inflammation and Endothelial Activation Makers after HCV Eradication with DAAs Therapy

All HIV/HCV patients achieved a sustained virological response (HCV RNA undetectable 12 weeks after the end of treatment, SVR). In parallel with HCV eradication, levels of liver enzymes significantly decreased (*p* < 0.0001 for the comparison between pre-DAAs and post-DAAs levels for all enzymes), returning to normal levels in all patients in whom levels were above the normal range at baseline. In concordance with this, the APRI score and the FIB-4 index also significantly diminished (*p* < 0.001 and *p* = 0.006, respectively), and returned to levels similar to those found in the HIV group. A slight increase of total cholesterol and LDL was also observed. Interestingly, the CD4 counts significantly increased (CD4 counts: 735 (577–902) cells/µL and 839 (618–1049) cells/µL at pre-DAAs and post-DAAs, respectively, *p* = 0.007) (Table S2). In contrast, in the HIV group, during a similar observation period (median: 9 (6–12) months), no significant change of CD4 counts was observed (CD4 counts: 816 (605–992) cells/µL and 821 (657–994) cells/µL at baseline and after 9 months, respectively, *p* = 0.225). Regarding liver stiffness (LS) values after HCV eradication, the median period of time between the baseline measurement and the post-treatment measurement was 25 (12–29) months. LS significantly decreased after this period of time (*p* = 0.002) (Table S2), with a delta of LS (difference between the post-DAAs and the pre-DAAs treatment measurements) of −1.0 (−3.0, −0.6) KPa, and all patients presented LS values <7.1 KPa.

**Figure 3:**
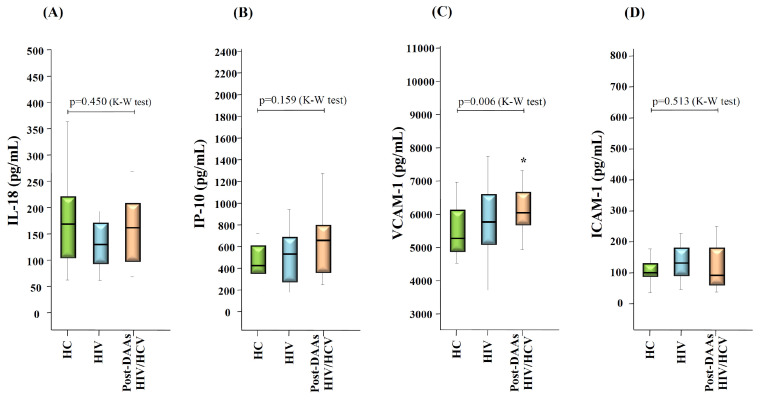
Box-plots graphs showing the levels of IL-18 (**A**), IP-10 (**B**), VCAM-1 (**C**) and ICAM-1 (**D**) in HC donors, HIV mono-infected, and HIV/HCV co-infected patients at 12 weeks after the end of DAAs therapy (post-DAAs HIV/HCV). *p*-values for the global comparison between the groups (Kruskal-Wallis test; K-W) are shown. (*): *p* < 0.05 with respect to the HC group (Mann-Whitney U test).

Twelve weeks after the end of treatment with DAAs (SVR time-point), there were significant decreases in the levels of IL-18 (*p* = 0.04), IP-10 (*p* = 0.001), VCAM-1 (*p* < 0.0001) and ICAM-1 (*p* = 0.003), compared with baseline levels. At this time-point, levels of IL-18 and ICAM-1 in the post-DAAs HIV/HCV group were comparable to those observed in the HIV group and in HC donors, whereas VCAM-1 levels remained significantly increased in the post-DAAs HIV/HCV group compared to HC donors (*p* = 0.006 for the global comparison and *p* = 0.001 for the pair-wise comparison between the post-DAAs HIV/HCV group and HC donors), and levels of IP-10 showed a trend (*p* = 0.13 for the global comparison and *p* = 0.07 for the pair-wise comparison between the post-DAAs HIV/VCV group and HC donors) (Figure 3).

**Figure 4:**
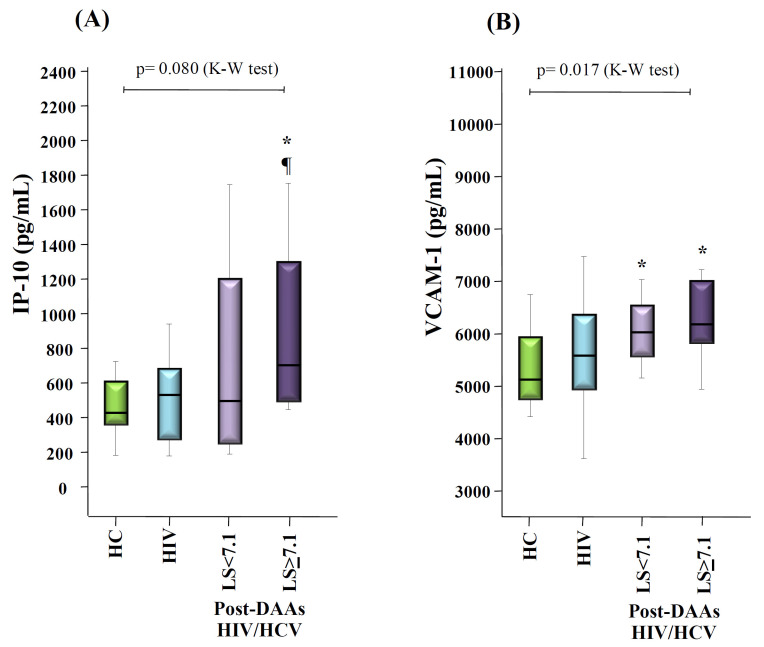
Box-plots graphs showing the levels of IP-10 (**A**), and VCAM-1 (**B**), in HC donors (HC), HIV monoinfected (HIV), and HIV/HCV coinfected patients at 12 weeks after the end of DAAs therapy (post-DAAs HIV/HCV), stratified according to the level of liver stiffness (LS) at baseline (before DAAs treatment). *p*-values for the global comparison between the groups (Kruskal-Wallis test; K-W) are shown. (*): *p* < 0.05 with respect to HC donors (Mann-Whitney U test). (¶): *p* < 0.05 with respect to the HIV group (Mann-Whitney U test).

Interestingly, after stratifying post-DAAs HIV/HCV patients according to their value of baseline liver stiffness (LS), in those patients with LS ≥ 7.1 KPa, the levels of IP-10 remained significantly increased after HCV eradication, compared to the HIV group and to the HC donors (*p* = 0.080 for the global comparison; *p* = 0.049 and *p* = 0.006 for the pair-wise comparisons with the HIV group and HC donors, respectively), and the levels of VCAM-1 remained significantly increased, compared to the HC donors (*p* = 0.017 for the global comparison; *p* = 0.006 for the pair-wise comparison) (Figure 4). In contrast, after HCV eradication all markers were normalized in post-DAAs HIV/HCV patients with LS < 7.1 KPa, except for VCAM-1 that remained significantly increased, compared to the HC donors (*p* = 0.005) (Figure 4).

## 4. Discussion

This longitudinal study examined the effect of HCV on relevant inflammatory, endothelial activation and coagulopathy bio-markers in a cohort of PLWH on cART, as well as the evolution of these markers after HCV eradication with the current DAAs therapy. The main findings of our study are: (a) HCV coinfection in PLWH significantly impacts on several bio- markers associated to inflammation and endothelial activation; (b) The alterations of these markers in HIV/HCV coinfected patients are more marked in patients with increased liver stiffness; (c) the eradication of HCV with DAAs-based therapy does not completely revert these alterations, at least in the short term.

HIV/HCV coinfected patients presented significantly higher levels of systemic inflammation and endothelial activation markers, including IL-18, IP-10, VCAM-1 and ICAM-1, in agreement with previous studies in HIV/HCV coinfected [12,14,16,22,23,24,25] and in HCV monoinfected [15,26,27] patients, suggesting a direct role for HCV in systemic inflammation and endothelial activation in the setting of HIV infection.

IL-18 is well known as a pleiotropic cytokine with potent proinflammatory activity [28]. It has been shown that HCV induces the production of IL-18 by human macrophages [29] and that necroinflammatory activity in the liver is associated with increased levels of IL-18 [26,27], which is in agreement with our findings showing a significant correlation of IL-18 levels with the levels of both HCV replication and liver enzymes. Thus, the ability to produce IL-18 in response to HCV replication could modulate the level of necroinflammatory activity in the liver, and the progression of liver disease, as has been previously proposed [27].

IP-10, also known as CXCL-10 chemokine, has also an important role in immune-pathogenesis of HCV infection, given its capacity to recruit several immune cells to the liver tissue [30,31]. Both expression in the liver tissue [30,32] and plasma levels [33] of IP-10 have been associated to necroinflammatory activity and progression of liver disease. Interestingly, in our study, levels of IP-10 were independently associated with GGT enzyme, a marker of liver damage and colestasis, but also of many other pathological conditions associated with an inflammatory state, such as cardiovascular disease, metabolic syndrome and cancer [34].

VCAM-1 and ICAM-1, markers of endothelial activation, are the molecules that facilitate the adhesion and recruitment of leukocytes to the vessel wall [35]. It has shown a direct effect of HCV-RNA on endothelial activation and upregulation of cell adhesion molecules on endothelial cells [36], which is in agreement with our findings of increased levels of VCAM-1 and ICAM-1 in HIV/HCV coinfected patients, compared to HIV monoinfected patients. Moreover, there was a significant correlation between VCAM-1 levels and AST levels, which suggests that necroinflammatory activity in the liver and endothelial activation are linked phenomena in the setting of HIV/HCV coinfection. Interestingly, increased levels of endothelial activation markers ICAM-1 and VCAM-1 have been associated with cardiovascular disease in both the uninfected [37] and in the HIV-infected population [38]. Thus, the increased levels of these markers in HIV/HCV coinfected patients could be related to the higher incidence of cardiovascular disease in HIV/HCV coinfected patients, compared to HIV monoinfected patients [39].

Levels of the inflammatory makers IL-18 and IP10, and levels of the endothelial activation marker ICAM-1 were positively correlated with levels of liver stiffness measured by transient elastography in HIV/HCV coinfected patients. This finding is in agreement with previous studies showing an association of different inflammatory and/or endothelial activation markers with the extent of liver fibrosis [13,16,25]. However, not all studies have found such an association [15,22,23] which may be due to, among other reasons, the different assays used to estimate the degree of liver fibrosis, either by using surrogate markers of fibrosis [13,15,16,23], or by direct histological assessment in liver biopsy [22,25]. It is interesting that, in our study, the significant association between plasma markers and liver stiffness in the univariate analysis disappeared after adjusting by the levels of liver enzymes in the multivariate linear regression analysis. Notably, levels of liver stiffness were associated with levels of liver enzymes, and with both the APRI score and the FIB-4 index. Taken together, these results suggest that, in our cohort of patients, levels of liver stiffness likely reflect the degree of hepatic injury more than the extent of liver fibrosis, since increased liver stiffness has also been associated with liver necroinflammation without fibrosis [40].

All HIV/HCV coinfected patients achieved SVR at 12 weeks after end of treatment. At this point, liver enzymes returned to normal levels (in all patients in whom levels of liver enzymes were increased at baseline], and the values of the APRI score and the FIB-4 index significantly diminished, in agreement with previous studies [41,42], suggesting a rapid improvement in hepatic injury after HCV eradication. We also observed a reduction of liver stiffness (LS] and all patients presented values < 7.1 KPa after a mean period of 25 months after treatment initiation. Thus, we could not verify if this decrease in LS values paralleled the rapid decrease of liver enzymes, which would support the link between LS and liver enzymes. Interestingly, other authors have found significant decreases of LS early after HCV eradication with DAAs therapy (12 weeks after end of treatment], in parallel with the normalization of liver enzymes [41,42], which supports an influence of necroinflammatory activity on LS values [40]. An interesting finding in our study was the significant increase in CD4 counts after HCV eradication in a relatively short period of only 12 weeks after the end of treatment, which supports a negative impact of HCV on immune restoration in HIV patients on ART, as has been previously suggested [43].

Our data showed a significant reduction of IL-18, IP-10, VCAM-1 and ICAM-1 plasma levels in HIV/HCV coinfected patients after HCV eradication with DAAs therapy. Only two previous studies have addressed this issue [18,23], although there are important differences in our study. First, Griesbeck et al. studied only four HIV/HCV patients receiving DAAs therapy, and analyzed a very limited number of plasma markers. Second, López-Cortés et al. [18], analyzed a large population of HIV/HCV coinfected patients, but DAAs regimens included ribavirin, and their study was focused on microbial translocation markers. Thus, ours is the first study analyzing the evolution of a large panel of plasma markers, including relevant inflammatory, endothelial activation and coagulopathy bio-markers, in a well-defined population of HIV/HCV coinfected patients before and after HCV eradication with DAAs therapy, and comparing with HIV monoinfected patients and with healthy controls. Interestingly, although all four bio-markers experienced significant decreases in HIV/HCV coinfected patients after DAAs therapy, in agreement with previous studies performed with cohorts of HCV monoinfected patients [44,45,46,47], they behaved in a different way after HCV eradication. Levels of IL18 normalized after DAAs treatment compared to the control group, in agreement with some previous studies in HCV monoinfection [47] but not with others [46], and the same was true for ICAM-1. In contrast, IP-10 and VCAM-1 remained significantly increased 12 weeks after the end of treatment with DAAs when compared with the control group. Increased levels of IP-10 after HCV eradication with DAAs has already been reported in HCV monoinfected patients [44,46,47], and thus, ours is the first study showing this in HIV/HCV coinfected patients. Our findings show that, despite liver inflammation regression after HCV eradication (as indicated by normalization of liver enzymes], both systemic inflammation and endothelial activation markers are not completely restored after HCV eradication, mainly in those patients with increased liver stiffness at the start of DAAs therapy, which may simply be the consequence of higher values of these markers at baseline in the subgroup of patients with increased liver stiffness. However, the evaluation of these markers was performed only 12 weeks after the end of treatment, and thus, further studies with longer follow up after HCV cure are needed, to ascertain the kinetics of normalization for these markers, and whether increased levels of IP-10 and VCAM-1 in the long term, in spite of HCV cure, could predict future liver or non-liver clinical events, as has been previously proposed for some of them [48].

Some aspects must be taken into account for the correct interpretation of our results. First, the relatively small sample size of our study groups, which limits the statistical power, and may preclude finding more subtle differences between the groups in the parameters analyzed. Second, our HIV/HCV coinfected cohort was not representative of the full spectrum of LS values, including only patients with moderately high LS values, and thus, the results and conclusions may be different for patients with very high values of LS. Third, we did not perform LS measurements in the group of HIV monoinfected patients, and thus, the existence of increased LS values cannot be ruled out, since other conditions apart from chronic HCV infection, such as high alcohol intake, or non-alcoholic fatty liver disease (NAFLD), as well as the body-mass index (BMI), may influence liver stiffness. However, no evidence of these conditions (high alcohol intake or NAFLD) was found in the medical records of any patient included in the study, and there were no differences in the BMI between the HIV and HIV/HCV groups.

## 5. Conclusions

In summary, our results showed that active HCV infection significantly impacts on systemic inflammation and endothelial activation markers, especially in those patients with increased levels of liver stiffness, suggesting that both active HCV replication and liver stiffness (as a surrogate of hepatic injury) influence the level of systemic inflammation and endothelial activation in the setting of HIV/HCV coinfection. Further, the normalization of some of these markers was not achieved, in spite of HCV eradication with DAAs, at least in the short term. This data prompts anti-HCV treatment in all HIV/HCV coinfected patients at the earliest stages of liver damage to enhance the normalization of systemic inflammation and endothelial activation markers.

## Figures and Tables

**Table 1:** Characteristics at baseline of patients included in the study.

Characteristic	HIV Group (*n* = 25)	HIV/HCV Group (*n* = 25)	*p*-Value
Age (years)	48 (42–55)	44 (39–48)	0.11
Gender (% of males)	88%	100%	0.24
Time since HIV diagnosis (years)	9 (6–14)	7 (2–10)	0.07
Time on cART (years)	5 (3.5–7.5)	4 (2–9)	0.63
Time since HCV diagnosis (years)	NA	2 (1.5–5)	NA
CD4 count (cells/μL)	816 (605–992)	735 (577–902)	0.31
CD4/CD8 ratio	0.84 (0.56–1.34)	0.75 (0.60–1.09)	0.49
ALT level (IU/L)	32 (24–37)	74 (49–162)	**<0.0001**
AST level (IU/L)	27 (22–31)	58 (37–122)	**<0.0001**
GGT level (IU/L)	36 (22–54)	55 (25–119)	0.06
Total cholesterol level (mg/dL)	194 (162–227)	157 (125–177)	**<0.0001**
HDL level (mg/dL)	42 (37–50)	46 (33–50)	0.815
LDL level (mg/dL)	119 (93–146)	85 (71–104)	**0.001**
Triglycerides level (mg/dL)	138 (88–208)	95 (70–140)	0.050
Body Mass Index (BMI)	25.1 (22.7–26.8)	24.1 (23–24.2)	0.835
HIV transmission route (%)			1
Sexual	100%	96%	
Parenteral	0%	4%	
HCV-RNA (log copies/mL)	NA	6.1 (5.8–6.4)	NA
HCV genotype (%)			NA
1a	NA	60%	
1b	NA	12%	
4	NA	28%	
Liver stiffness (KPa)	NA	5.8 (4.5–7.9)	NA
<7.1 KPa (F0-F1) (%)	NA	60%	
≥7.1 KPa (F2-F4) (%)	NA	40%	
APRI score	0.33 (0.27–0.50)	0.68 (0.43–1.58)	**<0.0001**
FIB-4 index	1.12 (0.78–1.36)	1.31 (1.01–1.97)	0.123

cART: combination antiretroviral therapy; ALT: alanine aminotransferase; AST: aspartate aminotransferase; GGT: gamma glutamil transferase; HDL: high density lipoprotein; LDL: low density lipoprotein; HCV: hepatitis C virus. Data for continuous variables are given as median [interquartile range]. NA: not applicable. In bold *p*-values < 0.05.

**Table 2:** Linear regression models showing the variables significantly and independently associated with the baseline level of IL-18, IP-10 and VCAM-1.

Dependent Variable	Independent Variables	Percentage of Variation Explained by the Model (R^2^)		Regression Coefficient (β ± SD)	*p*-Value
		Individual	Accumulated		
IL-18	AST level (IU/mL)	0.517	0.517	0.71 ± 0.14	<0.0001
	HCV-RNA (Log cop/mL)	0.115	0.632	47.9 ± 18.3	0.016
IP-10	GGT level (IU/mL)	0.307	0.307	1.60 ± 0.45	0.002
	LS ≥ 7.1 KPa	0.186	0.493	509 ± 179	0.010
VCAM-1	AST level (IU/mL)	0.199	0.199	5.78 ± 2.42	0.025

## Supplementary Materials

The following are available online at https://www.mdpi.com/2076-393X/8/2/323/s1. Figure S1: Scatter-plots graphs showing the correlations between baseline levels of IL-18 (A), IP-10 (B), and ICAM-1 (C) with baseline (before DAAs treatment) levels of liver stiffness in HIV/HCV patients. Spearman‘s rank correlation coefficient and *p*-value are shown inside the graphs. Figure S2: Correlations at baseline of liver stiffness values (kPa) whit ALT (A), AST (B), and GGT (C) liver enzyme levels, and with APRI score (D) and FIB-4 index (E) in HIV/HCV patients. Spearman‘s rank correlation coefficient and *p*-value are shown inside the graphs. Figure S3: Scatter-plots graphs showing the correlations of baseline (before DAAs treatment) levels of IL-18 (A, B, C, D), IP-10 (E, F, G, H), VCAM-1 (I, J, K) and ICAM-1 (L, M), with baseline levels of liver enzymes (AST, ALT and GGT), APRI score, FIB-4 index and HCV-RNA plasma viral load. Spearman‘s rank correlation coefficient and *p*-value are shown inside the graphs. Table S1: Liver stiffness values measured by transient elastography and validation criteria of each measurement in HIV/HCV coinfected patients at baseline (pre-treatment) and post-treatment with DAAs. Table S2: Liver enzymes, lipid profile, CD4 count, liver stiffness, APRI score and FIB-4 index in HIV/HCV patients at baseline (pre-DAAs) and after DAAs treatment (post-DAAs).
