# Supplementary material for: Liver Stiffness Hinders Normalization of Systemic Inflammation and Endothelial Activation after Hepatitis C Virus (HCV) Eradication in HIV/HCV Coinfected Patients
Source: Vaccines (Basel). 2020 Jun 19;8(2):323. doi: 10.3390/vaccines8020323 (PMC7350227; doi:10.3390/vaccines8020323)
Supplement: Supplementary file 1 [file vaccines-08-00323-s001.zip › Figure S2.pdf]

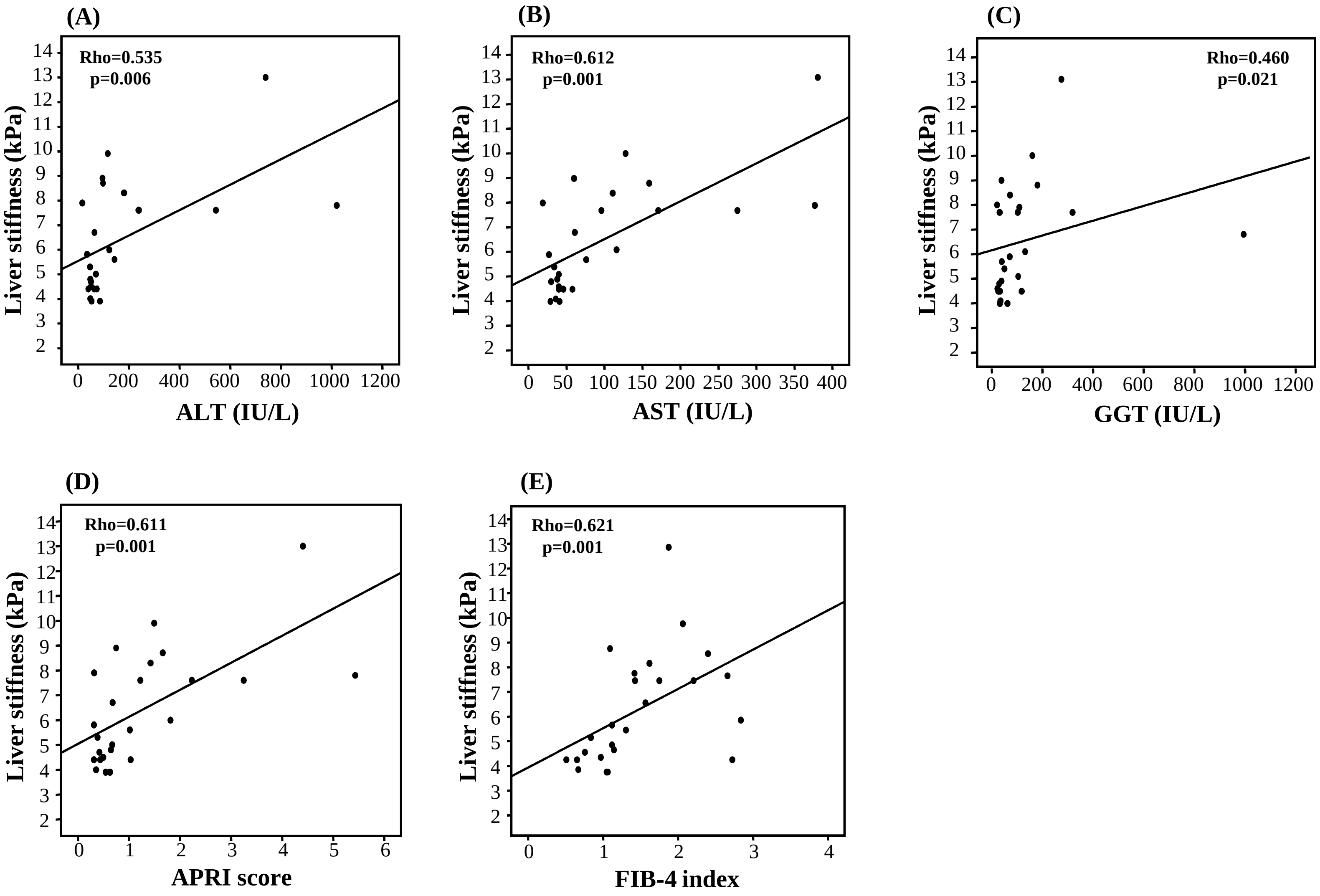

Figure S2. Scatter-plots showing the correlations between baseline (pre-DAAs treatment) liver stiffness values (kPa) with ALT (**A**), AST (**B**), and GGT (**C**) liver enzyme levels, and with APRI score (**D**) and FIB-4 index (**E**) in HIV/HCV patients. Spearman`s rank correlation coefficient and p-value are shown inside the graphs.
