# Supplementary material for: Liver Stiffness Hinders Normalization of Systemic Inflammation and Endothelial Activation after Hepatitis C Virus (HCV) Eradication in HIV/HCV Coinfected Patients
Source: Vaccines (Basel). 2020 Jun 19;8(2):323. doi: 10.3390/vaccines8020323 (PMC7350227; doi:10.3390/vaccines8020323)
Supplement: Supplementary file 1 [file vaccines-08-00323-s001.zip › Figure S3.pdf]

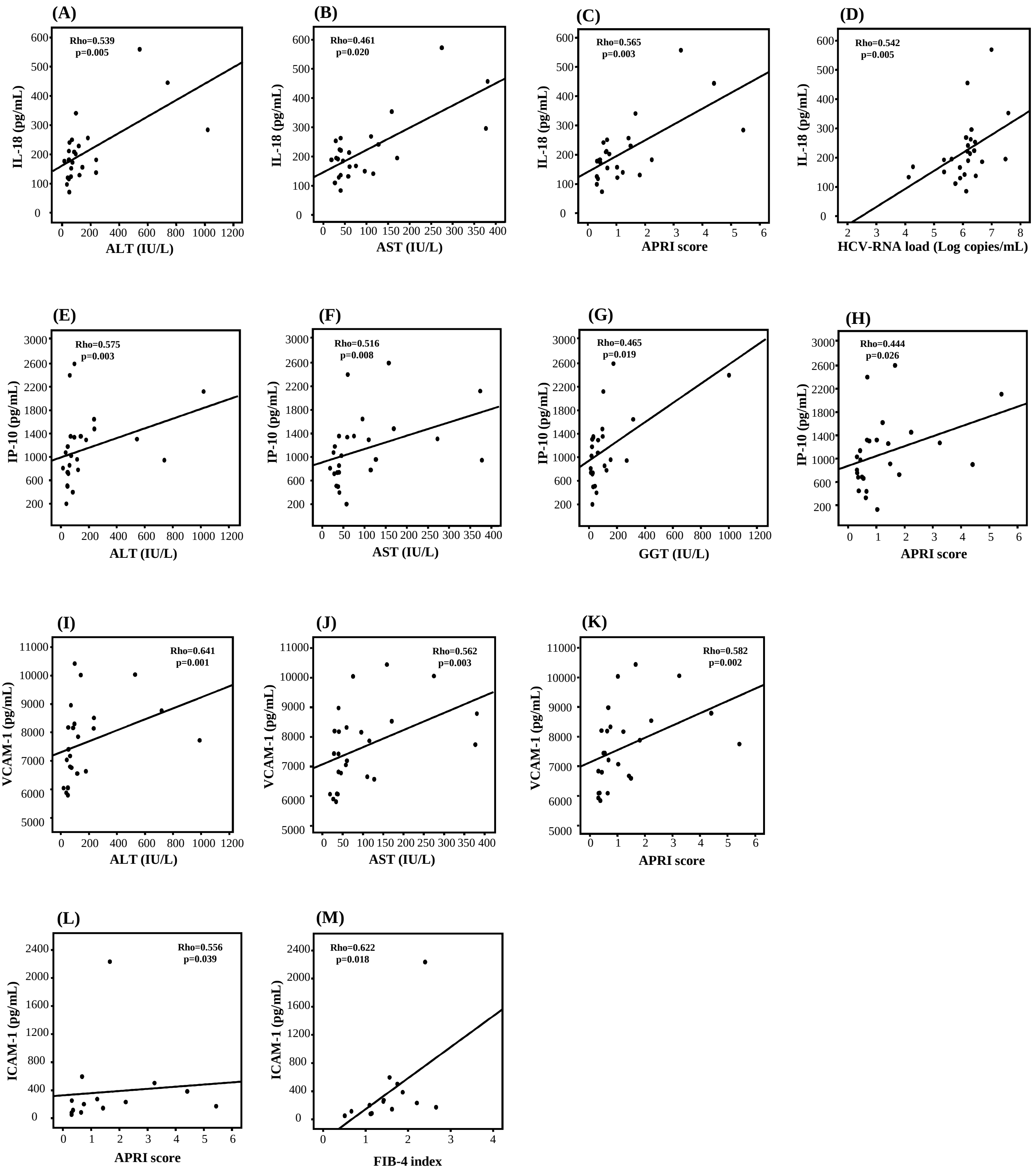

Figure S3. Scatter-plots showing the correlations between baseline (pre-DAAs treatment) levels of IL-18 (**A, B, C, D**), IP-10 (**E, F, G, H**), VCAM-1 (**I, J, K**) and ICAM-1 (**L, M**) with baseline levels of liver enzymes (ALT, AST and GGT), APRI score, FIB-4 index and HCV-RNA plasma viral load. Spearman`s rank correlation coefficient and p-value are shown inside the graphs.
