# Supplementary material for: Liver Stiffness Hinders Normalization of Systemic Inflammation and Endothelial Activation after Hepatitis C Virus (HCV) Eradication in HIV/HCV Coinfected Patients
Source: Vaccines (Basel). 2020 Jun 19;8(2):323. doi: 10.3390/vaccines8020323 (PMC7350227; doi:10.3390/vaccines8020323)
Supplement: Supplementary file 1 [file vaccines-08-00323-s001.zip › Figure S1.pdf]

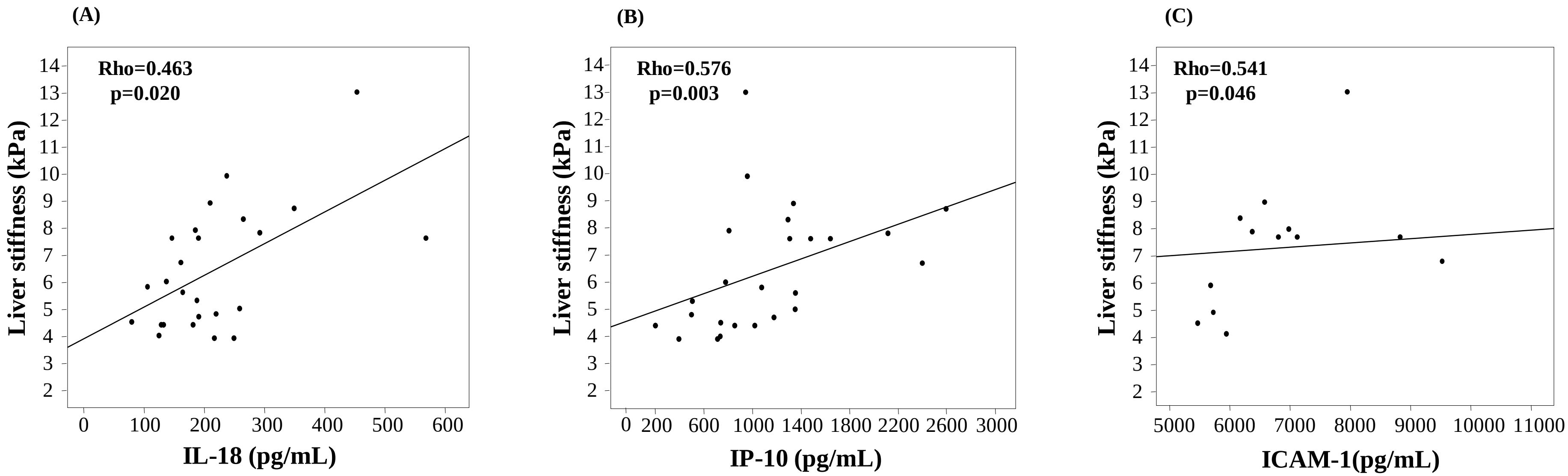

Figure S1. Scatter-plots graphs showing the correlations between baseline levels of IL-18 (**A**), IP-10 (**B**), and ICAM-1 (**C**) with baseline (before DAAs treatment) levels of liver stiffness in HIV/HCV patients. Spearman`s rank correlation coefficient and p-value are shown inside the graphs.
